# The Influence of Perceived Stress on Cortical Reactivity: A Proof-Of-Principle Study

**DOI:** 10.1371/journal.pone.0129220

**Published:** 2015-06-19

**Authors:** Rosan Luijcks, Catherine J. Vossen, Hermie J. Hermens, Jim van Os, Richel Lousberg

**Affiliations:** 1 Department of Psychiatry & Psychology, Maastricht University, Maastricht, The Netherlands; 2 Department of Anesthesiology and Pain Medicine, Maastricht University Medical Centre, Maastricht, The Netherlands; 3 Roessingh Research and Development, Enschede, The Netherlands; 4 King’s College London, King’s Health Partners, Department of Psychosis Studies, Institute of Psychiatry, London, United Kingdom; Wadsworth Center, UNITED STATES

## Abstract

The aim of this study was to investigate how perceived stress may affect electroencephalographical (EEG) activity in a stress paradigm in a sample of 76 healthy participants. EEG activity was analyzed using multilevel modeling, allowing estimation of nested effects (EEG time segments within subjects). The stress paradigm consisted of a 3-minute pre-stimulus stress period and a 2-minute post-stimulus phase. At t=3 minutes, a single electrical stimulus was delivered. Participants were unaware of the precise moment of stimulus delivery and its intensity level. In the EEG time course of alpha activity, a stronger increase was observed during the post-stimulus period as compared to the pre-stimulus period. An opposite time course effect was apparent for gamma activity. Both effects were in line with a priori expectations and support the validity of this experimental EEG-stress paradigm. Secondly, we investigated whether interaction effects of stress and coping, as measured with the Perceived Stress Scale-10 questionnaire (PSS-10), could be demonstrated. A higher perceived stress score was accompanied by a greater increase in delta- and theta-activity during the post-stimulus phase, compared to low scores. In contrast, low coping capacity was associated with a stronger decrease in slow beta, fast beta and gamma activity during the post-stimulus phase. The results of the present article may be interpreted as proof-of-principle that EEG stress-related activity depends on the level of subjectively reported perceived stress. The inclusion of psychosocial variables measuring coping styles as well as stress-related personality aspects permits further examination of the interconnection between mind and body and may inform on the process of transformation from acute to chronic stress.

## Introduction

Psychological and physical sequelae of stress are common. Experience of stress is accompanied by an increased level of arousal and may lead to a number of physiological reactions, such as acceleration of heart rate, pupil dilatation, increased galvanic skin response and increased finger pulse volume[1]. Increased muscle activity, especially in the trapezius muscles, and thus an increased EMG response is also associated with stress[2]. EMG activity can be viewed as a representation of peripheral stress, in the same way as brain activity may represent a central correlate of stress. Cortical activity in relation to stress can be studied using a range of dependent variables, such as electroencephalography (EEG), PET-scan or (f)MRI. In the present study, we chose to investigate the effects of experimentally induced stress on EEG activity. The main reason to focus on EEG as a stress-related variable is the possibility to model the dynamic time aspect of cortical activity.

A number of studies have investigated associations between EEG activity and stress [3–7]. Within these lines of research, in which stress has been experimentally manipulated, there is not only a focus on frontal alpha activity, but also on the left and right hemisphere asymmetry [3, 5, 8, 9]. It is assumed that the left hemisphere is more involved in the processing of positive emotions, whereas the right hemisphere is more involved in the processing of negative emotions[9]. Most studies report a relatively higher alpha activity in relaxation states compared to stressful situations [3, 5, 8, 10, 11]. In addition, stressful conditions like anxiety disorder [12] may be associated with alterations in EEG gamma activity[10, 13]. Subsequently, relatively lower alpha activity and relatively higher gamma activity can be expected during a stressful period. As to lateralization effects, since stress can be viewed as negative emotion, the effects of stress on the alpha and gamma band may be more pronounced in the right hemisphere.

Most stress experiments include a mental stress task. However, performing such a task may interfere with stress-related cortical processes in the EEG. The challenge in this type of research is to differentiate between cortical activity associated with performing the task itself and the cortical stress component. In response to this methodological problem, a novel experimental stress paradigm was recently developed. In this paradigm, one single unpredictable and uncontrollable electrical stimulus is presented, thus inducing both a distinct cognitive stressor and a physical stressful stimulus. The electrical stimulus divides the experimental phase into two periods: a pre- and a post-stimulus period. An anticipatory stress effect, a direct response to the nociceptive stimulus and a subsequent recovery to baseline levels were demonstrated for EMG activity[2]. In the latter study, it was demonstrated that EMG activity increased in the anticipatory pre-stimulus phase, followed by a decrease in EMG activity after the delivery of the stimulus. Additionally, it was shown that higher subjective stress was associated with a higher level of muscle activity. This new developed paradigm, validated with a subjective stress measure and with EMG, can be used to investigate the effects of experimentally induced stress on brain activity, examined by EEG.

The main goal of the present article is to further investigate the relationship between the bands of the full frequency EEG spectrum and experimentally induced stress. Differential cortical processing is expected between the anticipatory pre-stimulus period and the post-stimulus period of the novel design. Since stress is a phenomenon that can vary over time, stress-related EEG activity is expected to vary over time as well. Therefore, multilevel regression is required to model the within-condition EEG time effects. The average EEG effect of induced stress can be considered as a state, and gives no information about ongoing processes. The use of multilevel modeling provides insight in time-related EEG processing and may therefore lead to more informed conclusions. Thus, two a priori hypotheses were examined: (i) a relatively larger increase in alpha-activity and (ii) a relatively larger decrease in gamma activity were expected during the post-stimulus phase, compared to the stressful pre-stimulus phase. With respect to location effects, no a priori hypotheses were formulated, since these analyses pertain to the time course of the EEG activity, instead of absolute pre-post activity. In addition, a similar argumentation can be offered for lateralization effects.

Additionally, we were interested to investigate the interaction effect between subjective stress experience and the EEG time course. In the study on experimentally induced stress and its association with EMG activity[2], such a relation was demonstrated. It may be hypothesized that the level of perceived stress in the preceding weeks impacts brain activity during the experiment. This hypothesis is not only intuitive from a clinical point of view, but is also supported by experimental research [4]. In order to take this source of variation into account, the 10-item Perceived Stress Scale (PSS-10), comprising two subscales (stress and coping), was used[14].

## Materials and Method

### Ethics Statement

The study was conducted according to the principles of the Declaration of Helsinki and was approved by the ethics committee of the Academic Hospital Maastricht and Maastricht University (METC azM/UM, Maastricht). Before the start of the experiment, subjects provided written informed consent.

### Subjects

Seventy-six right-handed subjects (46 females and 30 males) participated in the study. Their age ranged from 18 to 65 years. Exclusion criteria were structural use of antipsychotics, anti-epileptics or anxiolytics during the past year or structural use of alcohol (>10 u/day). Subjects were asked to refrain from alcohol-containing consumptions the evening before and to refrain from caffeine-containing consumptions three hours prior to the experiment.

### Electroshocker and stimuli

An electro-shocker (type Shocko-100-AA-20, developed by Maastricht Instruments BV and approved for usage in experimental studies) was used to deliver electrical stimuli (see also [15]). Stimuli were electrical pulses of 10 milliseconds duration, administered intracutaneously on the top of the middle finger of the non-dominant left hand, as described by Bromm and Meier[16]. The sensation and pain threshold were determined by gradually increasing the intensity of the stimulus, starting at zero intensity. The first intensity that was consciously experienced was defined as the sensation threshold, the first intensity experienced as painful was defined as the pain threshold. This procedure was repeated three times in order to obtain a reliable estimate. The intensity of the electrical stimulus applied during the experiment was computed for each subject individually. The intensity of the actually delivered stress stimulus during the experiment was calculated as follows:

Actuallydeliveredstressstimulus=painthreshold+0.25*(painthreshold-sensationthreshold)

As shown in a previous experiment, this intensity level was experienced as painful by all subjects, albeit still acceptable [15].

### Procedure

EEG-electrodes as well as the shock electrode were attached, as described below. Next, a baseline measurement of 3 minutes was conducted. After determination of the individual pain threshold, subjects were instructed that they would receive a single electrical shock over a 5 minute period. The experimenter pointed out that the precise moment of stimulus delivery and its intensity level would be determined by a personal computer. In addition, participants were told that stimulus intensity might vary between the sensation threshold and a level clearly above the pain threshold. Subjects were instructed to keep both hands on the table, palms down, and not to close their eyes during the whole measurement period. In fact, all subjects received the experimental stimulus on exactly t = 3 minutes. The whole procedure was controlled by the software program “Presentation 0.71” (Neurobehavioral Systems).

### Psychophysiological recordings

All recordings were conducted in an electrically and sound-shielded cubicle (7,1 m2). Ag/AgCl electrodes were placed on 14 different locations (Fz, F3, F4, Cz, C3, C4, Pz, P3, P4, T3, T4, Oz, O1 and O2), using the international 10–20 system[17]. Impedances were kept below 5 kΩ. A reference electrode was placed on each ear lobe. To control for vertical eye movements, an EOG electrode was placed 1 cm under the midline of each eye. A ground electrode was placed at Fpz. All electrodes were fixed using 10–20 conductive past. Brainvision BrainAmp Research Amplifier (Brain Products; sampling rate 1000Hz, resolution 0.1μV) was used for EEG recording.

### Offline dataprocessing

EEG was recorded online with 1000Hz sampling rate, using Brainvision 2.0. Data was offline filtered (bandpass 0.5–50 Hz) and segmented into epochs of 512ms, without overlapping segments and using a Hamming window followed by a Fast Fourier transformation (FFT). The EEG spectrum was divided into frequency bands, which were defined as follows: delta (1–4 Hz), theta (4–8 Hz), alpha (8–13 Hz), slow beta (13–20 Hz), fast beta (20–30Hz) and gamma (30–50 Hz). Epochs with EOG activity exceeding +100mV and -100mV were excluded from the analyses.

### Psychological measurements

The 10-item Perceived Stress Scale was used to assess subjective stress over the past month. The PSS-10 has been translated into different languages and its validity has been demonstrated in several populations [18–20]. The two-factor structure of the PSS-10 was used, since there is a conceptual difference between both factors: the stress factor and the coping factor [21].

Psychometric analyses were performed. Factor analysis showed a two-factor structure, consisting of a 6-item stress factor and a 4-item coping factor. Crohnbach’s alpha’s for these two factors were 0.811 and 0.812 respectively. The 4 items that accounted for the coping factor were inverted. Consequently, a high score for the coping factor means that the coping capacity is low.

### Statistical analysis

Because of the hierarchical structure of the current EEG dataset, consisting of epochs (level 1) that are clustered within individuals (level 2), multilevel regression analyses were performed. In all multilevel regression analyses, EEG activity served as the dependent variable. In order to obtain normality, all dependent variables, consisting of the different EEG bands at different locations, were log transformed. Epoch number and condition (pre- versus post-stimulus period), as well as the covariates age and sex, served as independent variables in a basic model. The predictor of main interest was the interaction between the time variable (epoch number) and condition.

In order to discover which covariance structure yielded the best fit for the dataset, various covariance structures were tested. The -2 log likelihood of different models was calculated in order to determine which statistical model would fit best. An autoregression (AR1) structure turned out to be significantly better (lowest -2 log likelihood) than that of its competitors, namely compound symmetry (CS) and Scaled Identity. Since the dataset of EEG activity is composed as a multilevel data file, consisting of consecutive epochs, each epoch is correlated with the previous epoch, which makes an autoregression model convenient. The AR1 structure was therefore used for all statistical analyses. All models were executed with a random intercept. All statistical analyses were performed using SPSS 21.0. P-values equal to or below 0.05 were considered to be statistically significant.

## Results

Due to protocol violations (subjects who closed their eyes during the experiment, subjects who did not follow the instructions), 7 subjects were excluded from the analyses, leaving n = 69 analyzable participants (40 females, 29 males). Age ranged from 18 to 65, with a mean age of 36 years. The scores on the PSS-10 ranged from 2 to 24 (with a maximum of 40 points). For all subjects, the difference between the pain and sensation threshold was calculated. The average value of the sensation threshold was 0.35 mA (SD = range 0.10 mA to 1.05 mA); the average pain threshold was 1.37 mA (SD = range 0.15 mA to 3,65 mA); the average of all calculated differences in pain threshold and sensation threshold was 1,02 mA (SD = range 0.05 mA to 3.2 mA).

### EEG time course effects within the stress experiment

**Fig 1:**
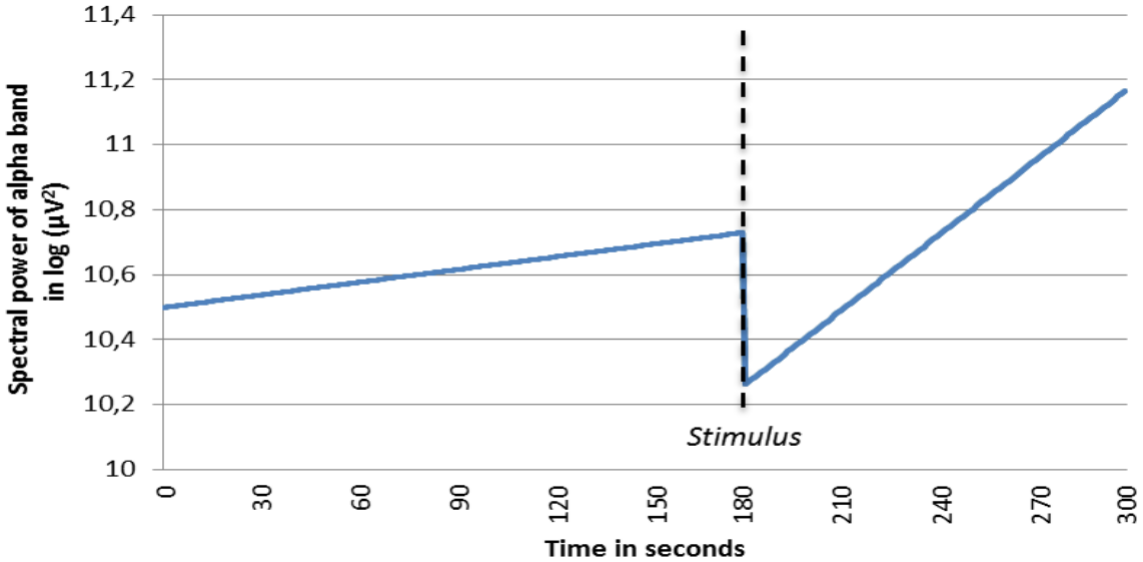
Fitted time course of spectral power of the alpha band for pre- and post-stimulus period. Not specified per location. Stimulus delivery took place on exactly t = 180 seconds. The increasing trend in spectral power is more apparent in the post-stimulus period compared to the pre-stimulus period.

**Fig 2:**
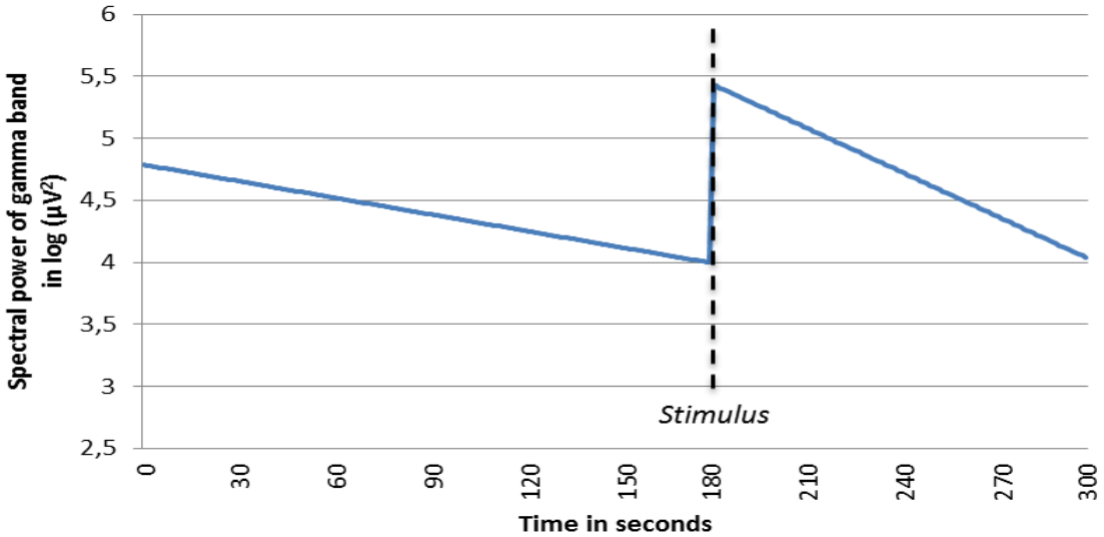
Fitted time course of spectral power of the gamma band for pre- and post-stimulus period. Not specified per location. Stimulus delivery took place on exactly t = 180 seconds. In both the pre- and post-stimulus period, a decreasing trend is present; nevertheless, a significant stronger decrease of gamma activity in the post-stimulus period is apparent.

Since no a priori hypotheses were formulated with respect to location effects, overall analyses of the spectral power time course were executed for all 6 EEG power bands in order to examine robust effects. For illustrational purposes, a graph of the predicted estimated EEG time course during the experiment was computed for the alpha and gamma activity band. With respect to the alpha activity, Fig 1 shows that the increasing trend in spectral power is more apparent in the post-stimulus period compared to the pre-stimulus period. For gamma activity, an opposite effect can be observed: in both the pre- and post-stimulus period, a decreasing trend is present (Fig 2). The significant negative t-value of -3.91 (as can be seen in Table 1) can be interpreted as a stronger decrease in gamma activity in the post-stimulus period. An overall analysis of the time*condition interaction was executed for all 6 EEG bands. As shown in Table 1, there was a significant positive interaction effect for the alpha and fast beta band, indicating a relatively stronger increase during the post-stimulus phase compared to the pre-stimulus phase. The significant negative interaction effect on the gamma band should be interpreted in the opposite way.

**Table 1:** Multilevel analyses of time*condition interactions on all 6 EEG-bands.

Band	T-value	P-value
**Delta**	-0,80	0.42
**Theta**	-0,49	0.62
**Alpha**	3,74	<0.01
**Slow beta**	4,18	<0.01
**Fast beta**	-0,33	0.74
**Gamma**	-3.91	<0.01

Values showed in Table 1 are T-values and p-values of the time*condition interaction effects. Condition was coded as 0 and 1, contrasting the pre- and post-stimulus period. Significant time*condition interaction effects were found for the alpha, slow beta and gamma band. The negative T-value for gamma activity can be interpreted as a stronger decrease in gamma activity in the post-stimulus period.

In addition, the same models were run for each of the 14 locations separately. As was expected from the (location) aggregated band analyses, for nearly none of the locations a time*condition interaction effect could be demonstrated on the delta and theta bands (Table 2). With respect to alpha and slow beta activity, the strongest effects were seen in the central and parieto-temporal area; no robust effects could be detected in the frontal and occipital area. Inspection of these results suggests a lateralization effect, the right side having stronger interaction effects than the left side. Additionally, for the fast beta band there are also significant interaction effects. However, there were both positive and negative interaction effects, which could be a reason for not finding an overall band effect (see previous paragraph). Finally, a significantly much higher significant decrease in gamma activity in the post-stimulus period compared to the pre-stimulus period was found, especially in the frontal, central and occipital regions.

**Table 2:** T-values for the time*condition interaction effects on all 6 EEG-bands, per location.

	Fz	F3	F4	Cz	C3	C4	Pz	P3	P4	Oz	O1	O2	T3	T4
**Delta**	-1,53	-1,49	-1,81	-0,59	-0,74	-0,57	-0,90	-0,48	0,70	-0,22	0,16	-0,06	-0,95	0,32
**Theta**	-1,80	-2,05*	-1,81	-0,30	-0,80	0,13	1,13	1,00	1,60	-0,47	-0,06	-0,23	0,35	1,37
**Alpha**	2,49*	0,65	1,44	4,06*	1,87	5,07*	4,75*	3,60*	5,53*	1,30	4,18*	2,85*	2,73*	3,95*
**Slow Beta**	3,97*	0,79	-0,19	5,90*	1,58	3,52*	5,81*	3,01*	5,78*	0,89	1,77	5,70*	4,50*	5,93*
**Fast Beta**	1,31	-2,13*	-2,99*	3,99*	-1,98*	-0,64	3,57*	-0,41	3,21*	-3,41*	-2,08*	2,62*	0,67	3,18*
**Gamma**	-4,26*	-4,67*	-5,80*	-2,56*	-4,31*	-3,26*	-1,89	-3,89*	-1,84	-4,46*	-2,89*	-0,02	-1,57	0,05

Values showed are T-values, demonstrating the significant time*condition interaction effects per location for all frequency bands. Condition was coded as 0 and 1, contrasting the pre- and post-stimulus period. Results with corresponding P-values ≤0.05 were considered to be significant (all significant results were marked with *). Negative T-values can be interpreted as a stronger decrease in EEG activity in the post-stimulus period.

### The effect of subjective stress on the EEG time course

For all EEG frequency bands, the interaction between the PSS-10 and the EEG time course during the experiment was examined, contrasting the pre-stimulus period and the post-stimulus period.

#### Stress factor

The stress component of the PSS-10 had a significant effect on the delta and theta condition*time interaction (Table 3). These results indicate that with a higher perceived stress score, a more pronounced increase in delta- and theta-activity exists during the post-stimulus phase when compared to the pre-stimulus phase.

**Table 3:** T-values for the time*condition*subjective stress interactions on all 6 EEG-bands.

Bands	PSS Coping		PSS Stress	
	T-value	P-value	T-value	P-value
**Delta**	-0.54	0.59	1.96	0.05
**Theta**	-1.83	0.07	2.74	0.01
**Alpha**	-1.80	0.07	-1.39	0.17
**Beta slow**	-2.31	0.02	0.96	0.34
**Beta fast**	-3.01	<0.01	-0.46	0.65
**Gamma**	-2.25	0.02	-1.16	0.24

Values showed are T-values and their corresponding p-values, demonstrating the time*condition interaction effects. Condition was coded as 0 and 1, contrasting the pre- and post-stimulus period. Subjective stress was defined by the two factors of the PSS-10: coping and stress. For both factors multilevel analyses were executed separately. Negative T-values can be interpreted as a stronger decrease in EEG activity in the post-stimulus period.

When specifying for location, taking into account all 14 locations, a frontal and central effect can be seen for theta activity (Table 4). Also, this PSS time course interaction effect appeared to be related to the left hemisphere (F3, C3, P3, O1, T3). There was a relatively strong PSS time course interaction effect on fast beta and gamma activity in the temporal area, as can be seen in Table 4.

**Table 4:** T-values for the time*condition*PSS stress interactions on all 6 EEG-bands, per location.

	Fz	F3	F4	Cz	C3	C4	Pz	P3	P4	Oz	O1	O2	T3	T4
**Delta**	2.87*	1.93	3.18*	1.93	1.46	2.47*	1.16	1.23	1.73	1.96*	1.16	1.43	1.66	1.85
**Theta**	3.05*	2.98*	2.46*	2.34*	2.85*	2.32*	1.68	2.30*	1.60	1.20	2.11	1.26	2.91*	1.95
**Alpha**	0.32	0.20	0.45	-1.35	-1.64	-0.45	-2.62*	-1.97*	-1.20	-0.88	-1.75	-1.30	-1.04	-0.88
**Beta slow**	1.86	2.24*	1.60	0.85	-0.31	0.86	-0.22	-0.58	0.71	1.31	1.60	1.96*	-2.29*	0.91
**Beta fast**	0.70	2.52*	1.34	-0.02	-1.73	0.57	-0.21	-2.87	0.65	1.35	0.67	-0.55	-3.70*	-2.72*
**Gamma**	1.31	2.16*	2.10*	0.20	-2.49*	-0.98	-1.31	-4.09*	-1.85	0.21	-1.38	-2.82*	-4.42*	-3.22*

Values showed are T-values, demonstrating the time*condition*PSS stress interaction effects per location for all frequency bands (significant results were marked with *). Condition was coded as 0 and 1, contrasting the pre- and post-stimulus period. Negative T-values can be interpreted as a stronger decrease in EEG activity in the post-stimulus period.

#### Coping factor

**Fig 3:**
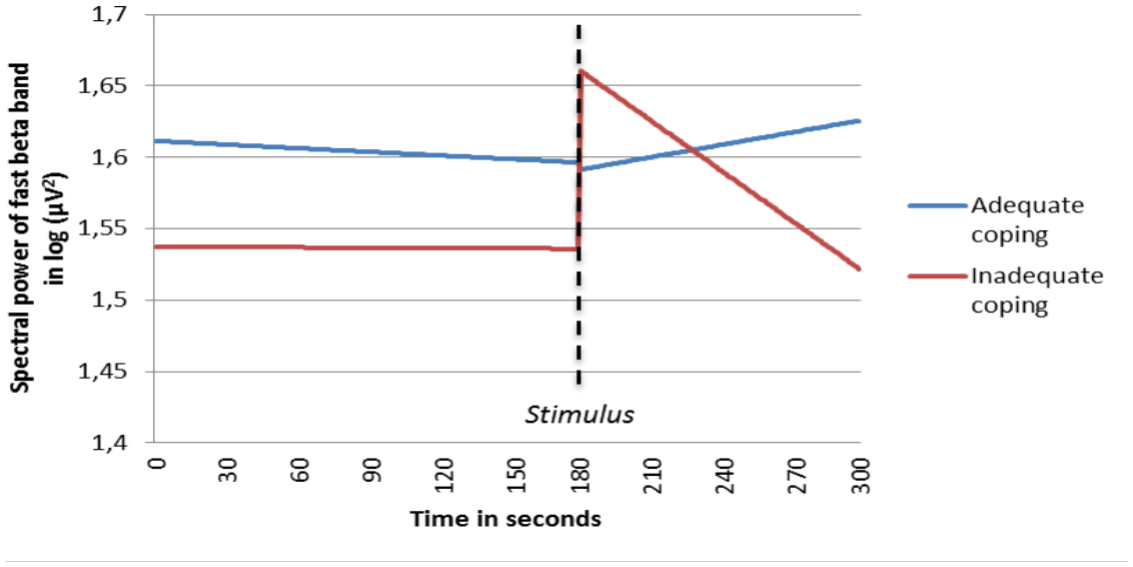
Fitted time course of spectral power of the fast beta band, interacted with coping capacity. Adequate coping and inadequate coping are contrasted. With a low coping capacity (i.e. high score on the questionnaire), a stronger decrease in time takes place in the post-stimulus period for spectral power of the fast beta band.

**Fig 4:**
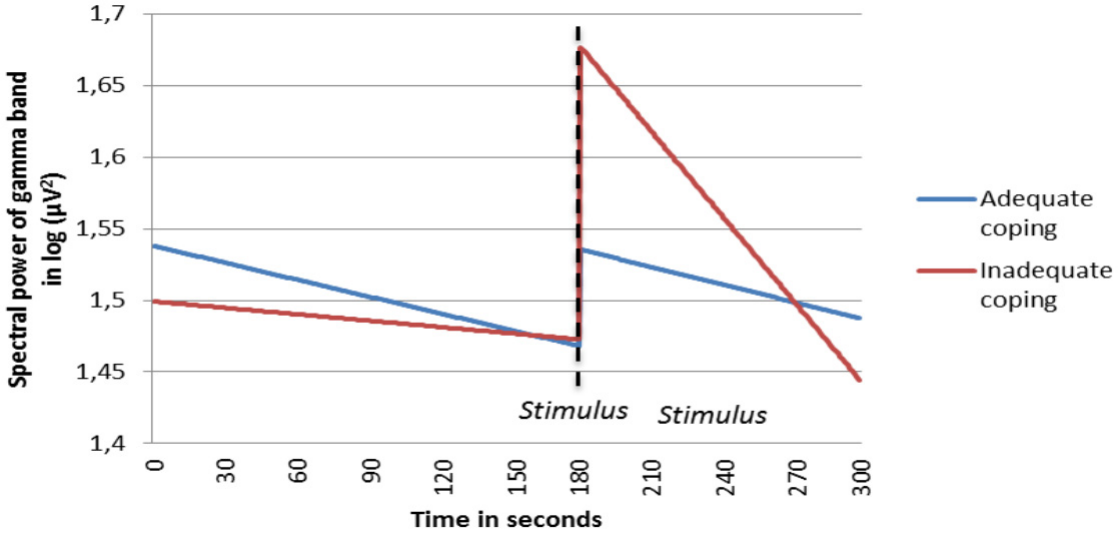
Fitted time course of spectral power of the gamma band, interacted with coping capacity. Adequate coping and inadequate coping are contrasted. With a low coping capacity (i.e. high score on the questionnaire), a stronger decrease in time takes place in the post-stimulus period for spectral power of the gamma band.

The coping factor of the PSS 10 shows a relation with slow and fast beta activity as well as with gamma activity. The results of the multilevel analyses show that with a low coping capacity (i.e. high score on the questionnaire), a stronger decrease in time takes place in slow beta, fast beta and gamma activity during the post-stimulus phase, in contrast with the pre-stimulus phase. Figures of the predicted estimated EEG time course were computed for the fast beta and gamma band (Fig 3 and Fig 4).

As shown in Table 3, the coping component of the PSS-10 has a significant effect on the time course of the complete beta band and the gamma activity. Table 5 depicts a specification for the 14 cranial locations. As can be seen, the time*coping interaction effect on slow beta has a trend on many locations, but this trend is only significant in parietal and temporal areas. On fast beta activity as well as on gamma activity there are significant effects on almost all locations. Additionally, there is also a clear significant effect on frontal alpha activity, which could not be demonstrated in the aggregated band analyses. It appears that with lower coping capacity alpha activity decreases more in the post-stimulus phase when compared to the pre-stimulus phase.

**Table 5:** T-values for the time*condition*PSS coping interactions on all 6 EEG-bands, per location.

	Fz	F3	F4	Cz	C3	C4	Pz	P3	P4	Oz	O1	O2	T3	T4
**Delta**	0.03	-0.95	0.76	0.90	-0.45	0.90	0.20	0.10	0.67	-0.36	-1.11	-0.18	-0.71	1.20
**Theta**	-2.00*	-1.24	-1.90	-1.56	-0.89	-1.50	-1.05	-0.39	-1.88	-1.94	-2.08*	-2.40*	-0.54	-1.52
**Alpha**	-2.52*	-2.37*	-2.19*	-1.80	-2.11*	-1.32	-1.14	-1.76	-0.90	-0.54	-1.60	-0.86	-1.91	-1.19
**Beta slow**	-1.60	-1.70	-1.72	-1.53	-1.80	-2.56*	-1.18	-2.22*	-2.68*	-0.55	-1.31	-1.54	-2.49*	-2.57*
**Beta fast**	-2.33*	-2.37*	-1.34	-1.23	-2.61*	-2.15*	-2.83*	-3.92*	-3.50*	-2.12*	-2.09*	-3.42*	-3.77*	-3.31*
**Gamma**	-2.62*	-2.19*	-0.62	-2.54*	-2.76*	-1.73	-2.09*	-3.00*	-2.45*	-0.98	-0.62	-2.99*	-3.34*	-2.72*

Values showed are T-values, demonstrating the time*condition*PSS coping interaction effects per location for all frequency bands (significant results were marked with *). Condition was coded as 0 and 1, contrasting the pre- and post-stimulus period. Negative T-values can be interpreted as a stronger decrease in EEG activity in the post-stimulus period.

## Discussion and Conclusions

This is, to the best of our knowledge, the first study investigating how the time course of stress-related EEG activity can be modeled using multilevel regression analysis. Based on evidence reported in experimental stress studies, we formulated expected directions of EEG time*condition interaction effects for alpha and gamma activity. Further, we examined whether and how subjective stress impacted these time*condition interaction effects, for both the stress factor and the coping factor of the PSS-10.

With respect to the EEG time course of alpha activity pre- and post-stimulus, alpha activity appeared to increase more during the post-stimulus period, than the pre-stimulus period, as a priori expected. This interaction effect may be considered obvious, since subjects were told that after experiencing one stimulus, no other stimulus would be delivered. The post-stimulus period can therefore be conceptualized as a phase in which the arousal effect from the induced stressor fades away.

A similar argumentation can be given for the a priori expected larger decreasing effect of gamma activity in the post-stimulus period. The decrease of gamma activity in the present experiment is consistent with findings in previous research, in which an increase in gamma activity was associated with experimentally induced stress in anxious people[8][22]. It is remarkable that most of the significant gamma effects were found in the frontal, central and temporal regions. In addition to the a priori hypothesized effects, an increasing effect in the slow beta band was observed. Before interpreting this effect, a next step would be to replicate this post-hoc finding.

The PSS-10 was used to investigate whether the EEG time course depends on perceived stress and coping capacity. No a priori hypotheses were formulated with respect to bands, locations and direction of time effects. Significant interaction effects between the PSS-10 subscales and the EEG time course were demonstrated. Interestingly, the coping and stress subscales showed different effects on the time course of spectral power. Concerning the stress factor of the PSS-10, a higher perceived stress score was accompanied by a greater increase in delta- and theta-activity during the post-stimulus phase, compared to the pre-stimulus phase. In contrast, the analyses of the coping factor of the PSS-10 showed that a low coping capacity was associated with a greater decrease in slow beta, fast beta and gamma activity during the post-stimulus phase. In case of aggregating all cranial locations, no interaction effects of the coping factor of the PSS-10 on the time course of alpha activity could be demonstrated. However, when analyzing all locations separately, significant effects on frontal alpha activity stand out.

The results of earlier studies can be brought to bear on the interpretation of the interaction effects between PSS and the time course of the spectral power of delta and theta bands. First, Klimesch et al. reported that increased theta activity is associated with the encoding of new information and recall of episodic memory[23, 24]. In these articles, it was demonstrated that the highest amount of relative theta power was apparent in the frontal and central areas. This is in line with the results of the present study. Second, it is known that stressful or emotionally arousing events can activate memory[25, 26]. Based on these findings, the following post hoc explanation is postulated: the increased theta-activity during the post-stimulus phase for people with a higher stress score may correspond to a recall of episodic memory, and thus to an evaluation of the stressor.

Another post hoc explanation is based on previous studies, in which the authors report an increase in delta and theta spectral power in the frontal area during a cold pressor test due to stress and painful activation [27, 28]. It was also implicated that heightened delta activity reflects the stress component of human pain responsivity in such a stress task[27]. Thus, the increase in delta and theta activity during the post-stimulus phase for people with a higher stress score may be related to this proposed mechanism. Although no a priori hypothesis was formulated, the interaction effect of the PSS coping factor and the EEG time effect on the alpha band in the frontal region (larger increase during the post-stimulus phase for subjects with a greater coping capacity) may be intuitively logical. Stated otherwise, in subjects with low perceived control, a state of relaxation after a stressor seems to be more difficult to attain, and is in line with results observed in other studies[4, 5, 29].

Since anxiety can be viewed as a form of stress, the results of some studies examining the relationship between anxiety and EEG activity are relevant. A relationship between anxiety and lower alpha activity in the prefrontal cortex is often suggested. The prefrontal cortex is directly connected with the amygdala, hippocampus and hypothalamus. The prefrontal cortex can inhibit limbic activity through these connections, as shown in human anxiety disorder studies[26, 30–32]. This mechanism may also apply to the present study, in which the interaction effect between stress coping and spectral power in the pre- and post-stimulus phase was examined. A clear effect was demonstrated in the frontal area for alpha activity, indicating that subjects with a lower coping capacity show a larger decrease in alpha activity. In other words, the impact of the acute stressor appears to be greater in subjects with lower coping capacity.

As to the significant effects observed in the other frequency bands, a clear post hoc explanation is lacking and replication of these findings is needed.

The generalizability of this particular stress experiment to a daily life stress situation may be a point of critique. However, we think that the uncontrollability and unpredictability of the stressor in the experimental paradigm are fundamental requirements of a stressful situation in general.

For future research, it would be interesting to use sLORETA analyses to further investigate regulatory mechanisms.

Since multilevel analysis is not frequently used in this area of research, relatively little knowledge on this topic has been gathered. The results of the present article should be interpreted as proof of principle regarding EEG changes during a stress experiment. Since stress reactivity is not a static but a dynamic process, multilevel analysis may be preferred to map out the EEG time course before, during and after a stressor. This statistical technique allows investigators to unravel psychophysiological response mechanisms in stress experiments. The fact that a different time effect was found for the anticipatory period and the post-stimulus period does plead for not merely examining main effects within stress experiments, but also investigating the time course. When focussing only on main effects, much of the information on underlying processes may not be revealed. Also, in case of a V-shaped mechanism or an inversed V-shaped mechanism, pre-post effects could be cancelled out and no main effect would be apparent.

In conclusion, this article demonstrates that with the current paradigm stress can be quantified with specific changes in EEG activity. Subsequently, the issue to be investigated is whether this reactivity depends on personal and environmental related variables. In this article the influence of perceived stress on cortical stress reactivity was shown. Including psychosocial variables measuring coping styles as well as stress-related personality aspects allows us to examine the interconnection between mind and body. This may especially be the case in stress research investigating the transformation process of acute stress to chronic stress.

Finally, it is important to mention the potential of this work in the rehabilitation of patients with a stress disorder, such as post-traumatic stress disorder. Since coping capacity is associated with health consequences later in life, it would be interesting to measure the electroencephalographic activity of these patients using the present stress experiment. If EEG-activity related to lower coping capacity could be objectified, early detection and preventive intervention of inadequate coping may be possible.

## Supporting Information

S1 Dataset Excel-file. (ZIP)
